# Voltage-Gated K^+^ Channel, K_v_3.3 Is Involved in Hemin-Induced K562 Differentiation

**DOI:** 10.1371/journal.pone.0148633

**Published:** 2016-02-05

**Authors:** Min Seok Song, Seon Young Choi, Pan Dong Ryu, So Yeong Lee

**Affiliations:** Laboratory of Veterinary Pharmacology, College of Veterinary Medicine and Research Institute for Veterinary Science, Seoul National University, Seoul, Korea; Federico II University of Naples, ITALY

## Abstract

Voltage-gated K^+^ (K_v_) channels are well known to be involved in cell proliferation. However, even though cell proliferation is closely related to cell differentiation, the relationship between K_v_ channels and cell differentiation remains poorly investigated. This study demonstrates that K_v_3.3 is involved in K562 cell erythroid differentiation. Down-regulation of K_v_3.3 using siRNA-K_v_3.3 increased hemin-induced K562 erythroid differentiation through decreased activation of signal molecules such as p38, cAMP response element-binding protein, and c-fos. Down-regulation of K_v_3.3 also enhanced cell adhesion by increasing integrin β3 and this effect was amplified when the cells were cultured with fibronectin. The K_v_ channels, or at least K_v_3.3, appear to be associated with cell differentiation; therefore, understanding the mechanisms of K_v_ channel regulation of cell differentiation would provide important information regarding vital cellular processes.

## Introduction

Voltage-gated K^+^ (K_v_) channels are well-established ion channels in excitable cells, where they serve as regulators of membrane potential and neuronal activities; however, these channels are also found in non-excitable cells, including cancer cells [1–3]. Previous studies have revealed cellular functions of K_v_ channels that include cell proliferation, apoptosis, and oxygen sensing [4–9]. Specifically, the modulation of certain K_v_ channel subunits, such as K_v_1.1, K_v_1.3, K_v_4.1, K_v_10.1, and K_v_11.1, significantly affects cancer cell proliferation [8, 10–13]. Nevertheless, even though a relationship is known to exist between cell proliferation and cell differentiation [14–16], a function for K_v_ channels in cell differentiation has not been well established. However, K_v_ channels may be involved in a series of cell differentiation mechanisms, and specific K_v_ channel subunits may have direct effects on cell differentiation.

K562 cells are human immortalized myelogenous leukemia cells obtained from the pleural fluid of patients with chronic myeloid leukemia in blast crisis [17]. These cells have been useful for studying hematopoietic cell proliferation and differentiation [18] and can differentiate into an erythroid lineage when treated with differentiation-inducing reagents such as hemin, sodium butyrate, and nicotinic acid [19, 20]. The induced cells produce hemoglobin, and differentiation can be validated by benzidine staining or hemoglobin quantification [18, 21–23]. K562 cells also can differentiate into megakaryotic lineages when treated with megakaryotic differentiation-inducing reagents, such as phorbol 12-myristate 13-acetate [24, 25].

K562 cell differentiation involves the mitogen-activated protein kinase (MAPK) and cAMP response element-binding protein (CREB) signaling pathways; extracellular signal-regulated kinase 1/2 (ERK1/2), CREB, and p38 have been specifically identified as important factors in K562 erythroid differentiation and hemoglobin synthesis [26–29]. In addition, certain K_v_ channels have close links to signaling molecules including CREB and CBP (CREB binding protein); they modulate K_v_ channel expression [30, 31].

Taken together, the available evidence suggests that K_v_ channels may be involved in the cell differentiation process through a range of signal pathways. An understating of the relationship between K_v_ channels and cell differentiation mechanisms might therefore suggest a new paradigm for cell differentiation research. In the present study, we investigated the roles of K_v_ channels and underlying signal mechanisms in the differentiation of K562 cells.

## Materials and Methods

### 2.1. Cell culture and hemin-induced cell differentiation

K562 cells obtained from Korean Cell Line Bank were cultured in RPMI1640 medium (Welgene, Daegu, Korea) supplemented with 10% fetal bovine serum (FBS) and 1% antibiotic-antimycotic solution (Sigma, St. Louis, MO) at 37°C incubation with 5% CO_2_. T25 flasks (SPL Life Sciences, Gyeonggi-do, Korea) were used for culturing the cells. When sufficient growth was achieved, 1 x 10^5^ cells were plated into a new T25 flask (SPL Life Sciences, Gyeonggi-do, Korea) and incubated with 50 μM hemin (Sigma, St. Louis, MO) to induce erythroid differentiation.

### 2.2. Reverse transcription-polymerase chain reaction (RT-PCR)

Total RNA was isolated using RNeasy Micro Kit (Quiagen, Valencia, CA) according to the manufacturer’s instructions. The cDNA was synthesized by reverse transcribing 1 μg of extracted RNA using random hexamers and an M-MLV reverse transcription kit (Promega, Madison, WI). The PCR reaction was performed with 2 μl of cDNA, 1× GoTaq^®^ green master mix (Promega), and target K_v_ channel specific primers using the following reaction conditions: initial denaturation at 94°C for 5 min, 35 cycles of cycling process (94°C for 40 s, the indicated annealing temperature (Table 1) for 40 s, 72°C for 1 min, and an extension at 72°C for 1 min), and a final extension at 72°C for 7 min (Table 1). All PCR products were subjected to electrophoresis on 1.6% agarose gel and analyzed using an ABI Prism 3730 XL DNA Analyzer (Applied Biosystems, Foster City, CA) to confirm their amplified sequences.

**Table 1:** RT-PCR primers.

Subtype	Accession No.	Size (bp)	Primer sequence (Forward/Reverse)	Annealing (°C)
K_v_1.1	L02750	498	5’-ACATTGTGGCCATCATTCCT-3’	55
			5’-GCTCTTCCCCCTCAGTTTCT-3’	
K_v_1.2	NM_004974.3	200	5'-ATGAGAGAATTGGGCCTCCT-3'	58
			5'-CCCACTATCTTTCCCCCAAT-3'	
K_v_1.3	NM_002232.3	177	5'-TGTCATGGCATCTCTTGC-3'	60
			5'-TGCATTTGGGATTCATTT-3'	
K_v_1.4	NM_002233.3	170	5'-ACGAGGGCTTTGTGAGAGAA-3'	58
			5'-GGTTTCCAGGCAAAAGATGA-3'	
K_v_1.5	M55513	917	5’-TGCGTCATCTGGTTCACCTTCG-3’	60
			5’-TGTTCAGCAAGCCTCCCATTCC-3’	
K_v_2.1	L02840	451	5’-GGAAGCCTGCTGTCTTCTTG-3’	65
			5’-CTTCATCTGAGAGCCCAAGG-3’	
K_v_3.3	AF055989	284	5’-CCTCATCTCCATCACCACCT-3’	60
			5’-CGAGATAGAAGGGCAGGATG-3’	
K_v_3.4	M64676	631	5’-TTCAAGCTCACACGCCACTTCG-3’	65
			5’-TGCCAAATCCCAAGGTCTGAGG-3’	
K_v_4.3	AF048712	349	5’-TGAGCTGATTGTCCTCAACG-3’	60
			5’-GTTCTCCGAGTCGTTGTCGT-3’	
K_v_9.3	NM_002252.3	200	5'-CAGTGAGGATGCACCAGAGA-3'	60
			5'-TTGCTGTGCAATTCTCCAAG-3'	

### 2.3. Western blotting

K562 cells were lysed with 1X passive lysis buffer (Promega, Madison, WI) and total protein was quantified with a BCA protein assay kit (Pierce, Rockford, IL). The extracted proteins were separated on a 10% SDS-PAGE and then transferred to Nitrocellulose membranes (Whatman, Maidstone, Kent). After blocking in 1x TBS-Tween 20 containing 5% nonfat milk (5% TTBS) (Difco Franklin Lakes, NJ), membranes were probed with specific antibodies (in 5% TTBS) for K_v_2.1, K_v_3.3, p38, phospho-p38 (Abcam, Cambridge, MA), K_v_1.2, K_v_1.3, CREB, phospho-CREB (Millipore, Billerica, MA), ERK, phospho-ERK (Cell Signaling Technology, Inc, Danver, MA), K_v_9.3, c-fos or β-actin (Santa Cruz Biotechnology, CA, USA). After overnight incubation, membranes were treated with horseradish peroxidase-conjugated goat, anti-rabbit secondary antibody (Santa Cruz Biotechnology, CA, USA) and visualized using an enhanced chemiluminescent detection kit (iNtron Biotechnology, Gyeonggi-do, Korea)

### 2.4. Real-time RT-PCR

A standard curve and primer efficiency were analyzed from the standard curve prepared from diluted cDNAs (2 or 10 fold) using a primer of GAPDH, a house keeping gene, as a reference. The real-time RT-PCR reaction was performed with 2 μl of cDNA, 1x SYBR Green Master Mix (Applied Biosystems, Foster City, CA), and 0.2 μM forward and reverse primers (Table 2) in the following reaction: initial step 95°C 30 s, then 40 cycles of the at 95°C for 5 s, and either 60°C (K_v_3.3) or 55°C (integrin) for 45 s. A dissociation protocol was used to confirm that paired primers produced only a single product. All of the procedures were performed using an Applied Biosystems StepOnePlus^™^ Real-Time PCR System (Applied Biosystems, Foster City, CA). The relative mRNA expressions of the K_v_ channel gene were normalized to the GAPDH gene and expressed as a fold change relative to the control group.

**Table 2:** Real-time RT-PCR primers.

fig	Accession No.	Size (bp)	Primer sequence (Forward/Reverse)	Annealing (°C)
K_v_2.1	NM_004975.2	173	5’-GTTGGCCATTCTGCCATACT-3’	60
			5’- GCAAAGTGAAGCCCAGAGAC-3’	
K_v_3.3	NM_004977.2	147	5’- CCTTCCTGACCTACGTGGAG-3’	60
			5’- CGAGATAGAAGGGCAGGATG-3’	
K_v_3.4	NM_004978.4	178	5’- AATATCCCAGGGTGGTGACA-3’	60
			5’- GGTCTTCAAAGCTCCAGTGC-3’	
K_v_9.3	NM_002252.3	200	5’- CAGTGAGGATGCACCAGAGA-3’	60
			5’- TTGCTGTGCAATTCTCCAAG-3’	
integrin β1	NM_002211.3	209	5’- CATCTGCGAGTGTGGTGTCT-3’	55
			5’- GGGGTAATTTGTCCCGACTT-3’	
integrin β3	NM_000212.2	176	5’- GCAATGGGACCTTTGAGTGT-3’	55
			5’- GTGGCAGACACATTGACCAC-3’	

### 2.5. Benzidine staining

Cultured cells were collected and centrifuged at 400 g for 5 min to obtain cell pellets. The cell pellets were washed using PBS and recentrifuged for 5 min. A benzidine working solution was prepared by mixing 20 μl of hydrogen peroxide solution (Sigma, St. Louis, MO) in 1 ml benzidine stock solution [0.2% 3,3′-dimethoxybenzidine (Sigma, St. Louis, MO) dissolved in 3% glacial acetic acid solution (Sigma, St. Louis, MO)]. The cell pellets were incubated at room temperature with the solution for 2 min and passed through the washing step again. Benzidine positive cells were analyzed by light microscopy. The K562 cells that were cultured in fibronectin coated wells underwent the washing and staining steps without centrifugation.

### 2.6. Hemoglobin quantification

Cell pellets were collected for protein extraction. Extracted protein was quantified using a BCA protein assay kit (Pierce, Rockford, IL). Quantitative analysis of hemoglobin was performed using a QuantiChromTM Heme Assay Kit (BioAssay Systems, Hayward, CA) following manufacturer’s instructions. The unit-hemoglobin contents (ng Hb/μg Protein) were calculated by dividing the amount of hemoglobin by the total amount of protein.

### 2.7. Patch clamp recordings

Poly-L-lysine coated 12 mm coverslips (SPL) was put into the recording chamber to allow the cells to be attached on the bottom of the chamber. K562 cells were centrifuged and then resuspended with bath solution to be transferred to the chamber. The cells were visualized by the differential interference contrast video microscopy (OLYMPUS, Tokyo, Japan). Patch pipettes were pulled from the borosilicate glass capillaries (1.7 mm diameter; 0.5 mm wall thickness) (World Precision Instruments, Sarasota, FL); the range of the seal resistance was from 8 to 10 MΩ. The internal pipette solution (in mM concentration) was consist of 135 K-gluconate, 5 KCl, 20 HEPES, 0.5 CaCl_2_, 5 EGTA, and 5 ATP-Mg and the bath solution is consist of 126 NaCl, 26 NaHCO_3_, 5 KCl, 1.2 NaH_2_PO_4_, 2.4 CaCl_2_, 1.2 MgCl_2_, and 10 glucose. The currents were recorded in the whole cell configuration by using Axoclamp 2B amplifier (Axon Instruments, Foster City, CA). Electric signal was filtered at 1 kHz and digitized at 10 kHz using analog-digital converter (Digidata 1320A, Axon Instruments) and pClamp software (Version 9.0, Axon Instruments). For the voltage-clamp mode, following protocol was used: cells were hyperpolarized by -90 mV pulse for 320 ms and the membrane currents were activated by depolarizing pulse for 400 ms from a holding potential -80 mV to the test potential which are ranged from -70 to 40 mV in 10 mV increments.

### 2.8. Inducing cell differentiation after transfection with small interference RNA (siRNA)

Cells were transfected with siRNA-K_v_3.3 using ON-TARGET plus SMART pool Human KCNC3 (Thermo Scientific Dharmacon, Lafayette, CO) and Lipofectamine^™^ 2000 reagent (Invitrogen, Carlsbad, CA, USA) following the manufacturer’s instructions for suspension cells. The ON-TARGET plus^®^ Control pool (Thermo Scientific Dharmacon, Lafayette, CO) was used as a control siRNA. The K562 cells (1 x 10^5^) were plated in 6 well plates immediately prior to the transfection step in RPMI 1640 (Welgene, Daegu, Korea) containing 10% FBS without any antibiotics. After 24 h, the siRNA-K_v_3.3 transfected cells were incubated with 50 μM hemin in 6 well plates. The incubation time was 24 or 48 h for benzidine staining or hemoglobin quantification, respectively. To induce cell adhesion, 6 well plates containing 10 μg/ml of fibronectin (Sigma, St. Louis, MO) dissolved in DPBS were incubated at 4°C for one day, and the coated plates were used for the experiment in place of uncoated plates.

### 2.9. K_v_3.3 overexpressed K562 cell line establishment

HEK293T cells (GE lifesciences) were maintained in 10% fetal bovine serum and 1% penicillin/streptomycin in Dulbecco’s modified Eagle medium (DMEM) at 37°C and 5% CO2. 24 hours before transfection, 6×10^6^ HEK293T cells were seeded into 100 mm dish. The following day, 50 μL (47.5 μg) of a Trans-lentiviral packaging mix encoding viral proteins Gag-Pol, Rev, and VSV-G and 42 μg of lentiviral transgene plasmids were transfected into each well for lentivirus production using Calcium phosphate. 14 hours after transfection, the DNA-reagent mixture was removed and replaced with 5% FBS in 14 ml fresh DMEM. At 48 hours post-transfection, lentiviral supernatants were harvested and filtrated with 0.45-μm filters. 1 volume of cold (4°C) PEG-it Virus Precipitation Solution (Systembio) was added to every 4 volumes of lentiviral particle-containing supernatant. The supernatant/PEG-it mixture was centrifuged at 1,500 x g for 30 minutes at 4°C. The viral pellet was resuspended and combined the lentiviral particles in 10 μL using cold (4°C), DMEM media. To determine transduction efficiency, HEK293T cells (1×10^3^, of 96-well plate) were transduced with lentivirus harvested from HEK293T cell transfection. At 72 hours post-transduction, GFP-positive cells were observed by fluorescence microscope (NiKon). Lentivirus was added to K562 cell culture in RPMI media supplemented with 10% FBS. Lentivirus was then added to give a multiplicity of infection (MOI) of 1. After overnight incubation, lentivirus was removed and fresh media added. K562 cells were then transduced with KCNC3 (Openbiosystem, OHS5898-202623948) construct and selected by 2 μg/ml Blastcidin to create K562-KCNC3.

### 2.10. Statistical analysis

All data are shown as means ± standard error (SE) and the Student *t*-test or One-way ANOVA was used to analyze the data (GraphPad Prism version 5.0).

## Results

### 3.1. Expression of K_v_ channels in K562 cells and the induction of K562 cell erythroid differentiation using hemin

**Fig 1:**
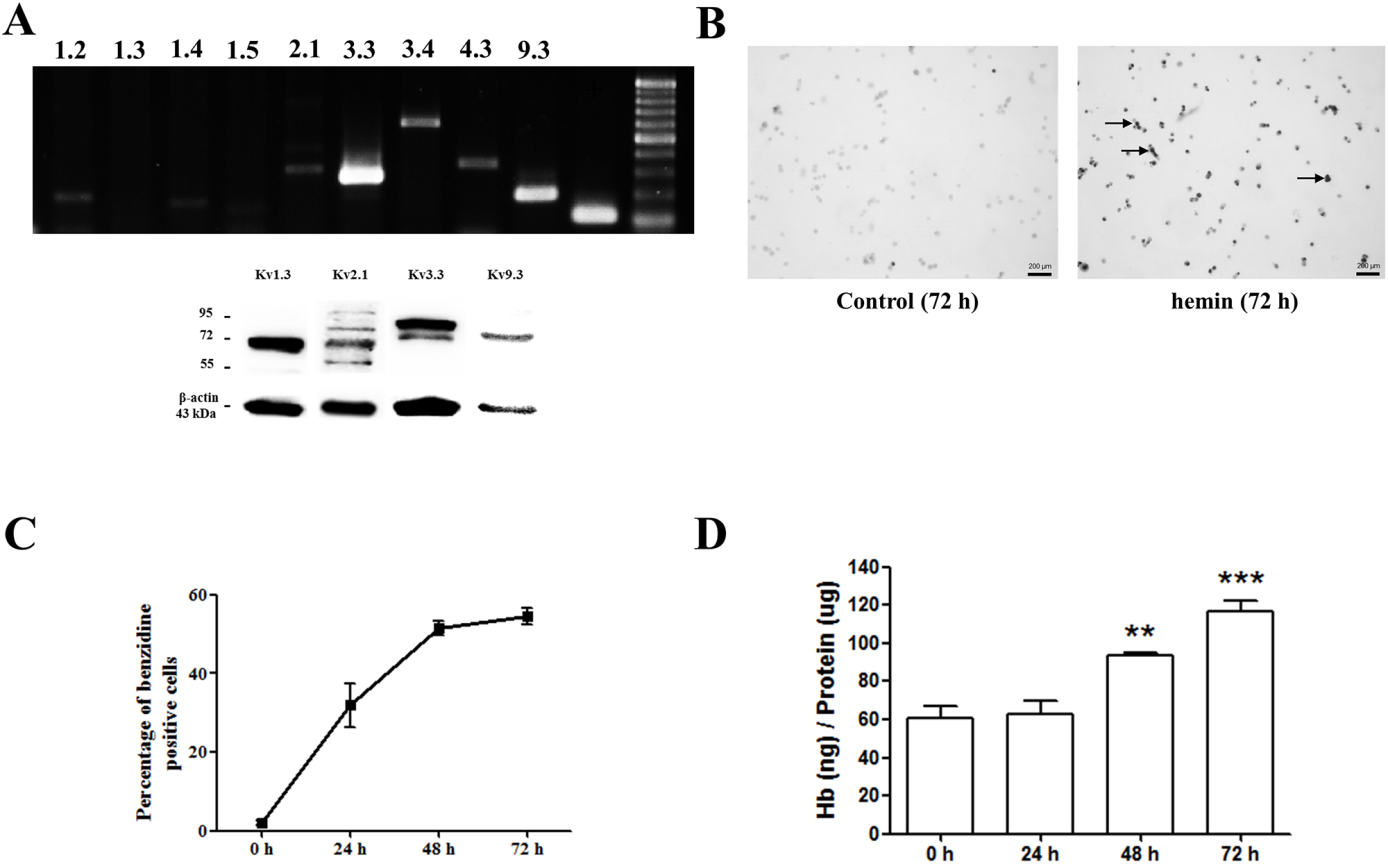
Identification of K_v_ channels in K562 cells and the erythroid differentiation of K562 cells using hemin. (A) RT-PCR data analysis demonstrated 7 different subtypes of K_v_ channels (K_v_1.2, K_v_1.4, K_v_1.5, K_v_2.1, K_v_3.3, K_v_3.4, K_v_4.3, and K_v_9.3). Western blot demonstrated the protein expression of K_v_1.3, K_v_2.1, K_v_3.3, and K_v_9.3. (B) K562 cells differentiated into erythroid cells were stained with benzidine after 72 h of differentiation using hemin (magnification ×40). Benzidine-positive cells appeared black, indicated by colored arrows. (C) The percentage of benzidine-positive cells was counted at 4 different time points (0, 24, 48, and 72 h). (D) The hemoglobin content of the differentiated K562 cells was measured at each indicated time point using a modified QuantiChrom Heme Assay. The concentration of hemoglobin at each time point was expressed as nanograms of heme per microgram of total protein.

RT-PCR analysis revealed that seven different K_v_ channels (K_v_1.2, K_v_1.4, K_v_2.1, K_v_3.3, K_v_3.4, K_v_4.3, and K_v_9.3) were detected in K562 cells (Fig 1A). K_v_1.1, K_v_1.3, and K_v_1.5 were also detected; however, the expression was too low. Western blot analysis demonstrated that K_v_2.1, K_v_3.3, K_v_3.4, and K_v_9.3 exist in K562 cells (Fig 1A). Hemin was used to induce K562 cell differentiation into erythroids (Fig 1B). Negative control data showed few stained cells. At an early stage of differentiation, the percentage of benzidine-positive cells, which were dyed black, rapidly increased and later reached a plateau (Fig 1C). The concentration of hemoglobin, on the other hand, showed no significant changes in the early stage of differentiation and started to increase between 24 and 48 h (Fig 1D).

### 3.2. The expression of K_v_ channels and whole cell patch clamp recording during the late stage of differentiation

**Fig 2:**
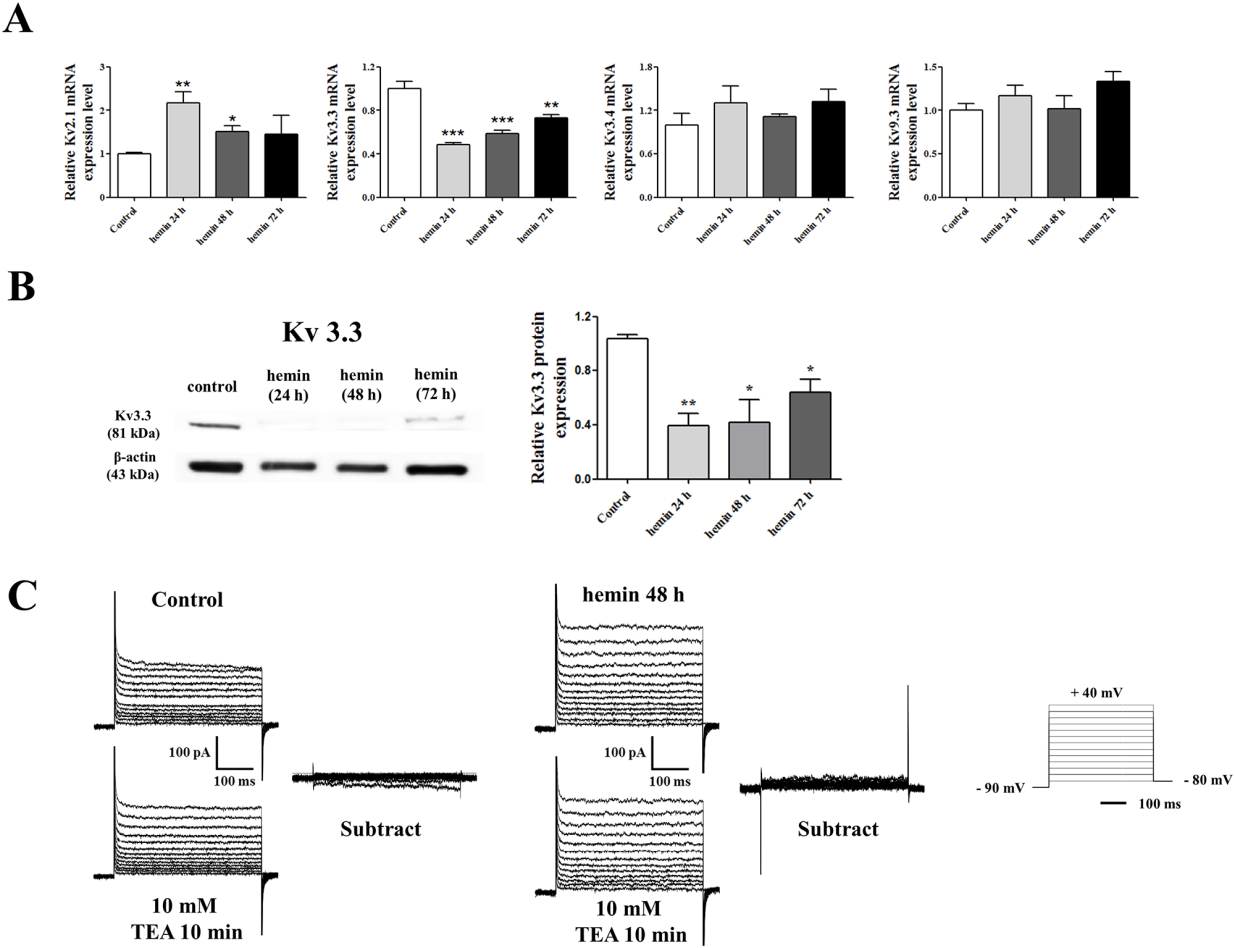
The expression of K_v_ channels and whole cell patch clamp recording during the late stage of differentiation. (A) After hemin-induced K562 cell differentiation, the mRNA expression levels of K_v_ channels, including K_v_2.1, K_v_3.3, K_v_3.4, and K_v_9.3, were compared with those of undifferentiated cells. The mRNA expression level of K_v_2.1 was increased, whereas the mRNA expression level of K_v_3.3 was decreased during erythroid differentiation. The mRNA expression of K_v_3.4 and K_v_9.3 were not altered during erythroid differentiation. The relative mRNA expressions of the Kv channels were normalized to the GAPDH gene and expressed as a fold change relative to the Mock control group. (B) The protein level of K_v_3.3 decreased significantly after 24 hours of hemin-induced erythroid differentiation in K562 cells. The relative protein expression of the K_v_3.3 was expressed as a fold change relative to the control group. (C) Representative current traces recorded from K562 cells. There was no TEA-sensitive current before and after hemin-induced erythroid differentiation. Experiments were performed in triplicate, and data are expressed as mean ± standard error. **p<0.01 compared with control value.

The real-time quantitative RT-PCR data demonstrated that the mRNA expression level of K_v_3.3 was decreased by half after 24 hours of erythroid differentiation and the reduction rate gradually slowed down, whereas K_v_2.1 increased during the K562 erythroid differentiation (Fig 2A). K_v_3.4 and K_v_9.3 showed no change and K_v_1 subunits were not detected (Fig 2A). The protein expression level of K_v_3.3 was also significantly lower during erythroid differentiation as a result of hemin treatment at the indicated time points (24, 48, and 72 h) (Fig 2B). We have tried to perform Western blot analysis for Kv2.1 in order to examine protein expression during differentiation; unfortunately, we could not obtain clear Western blot images for Kv2.1 in K562 cells. Therefore, we focused on Kv3.3 for the following experiments. Fig 2C demonstrated representative current traces recorded from K562 cells. Despite the fact that we detected the mRNA and protein expression of K_v_ channels, there was no TEA-sensitive current before and after hemin-induced erythroid differentiation (Fig 2C).

### 3.3. K_v_3.3 knockdown using siRNA-K_v_3.3 increased hemin-induced K562 cell differentiation, whereas K_v_3.3 overexpression did not decrease the hemin-induced K562 erythroid differentiation effectively

**Fig 3:**
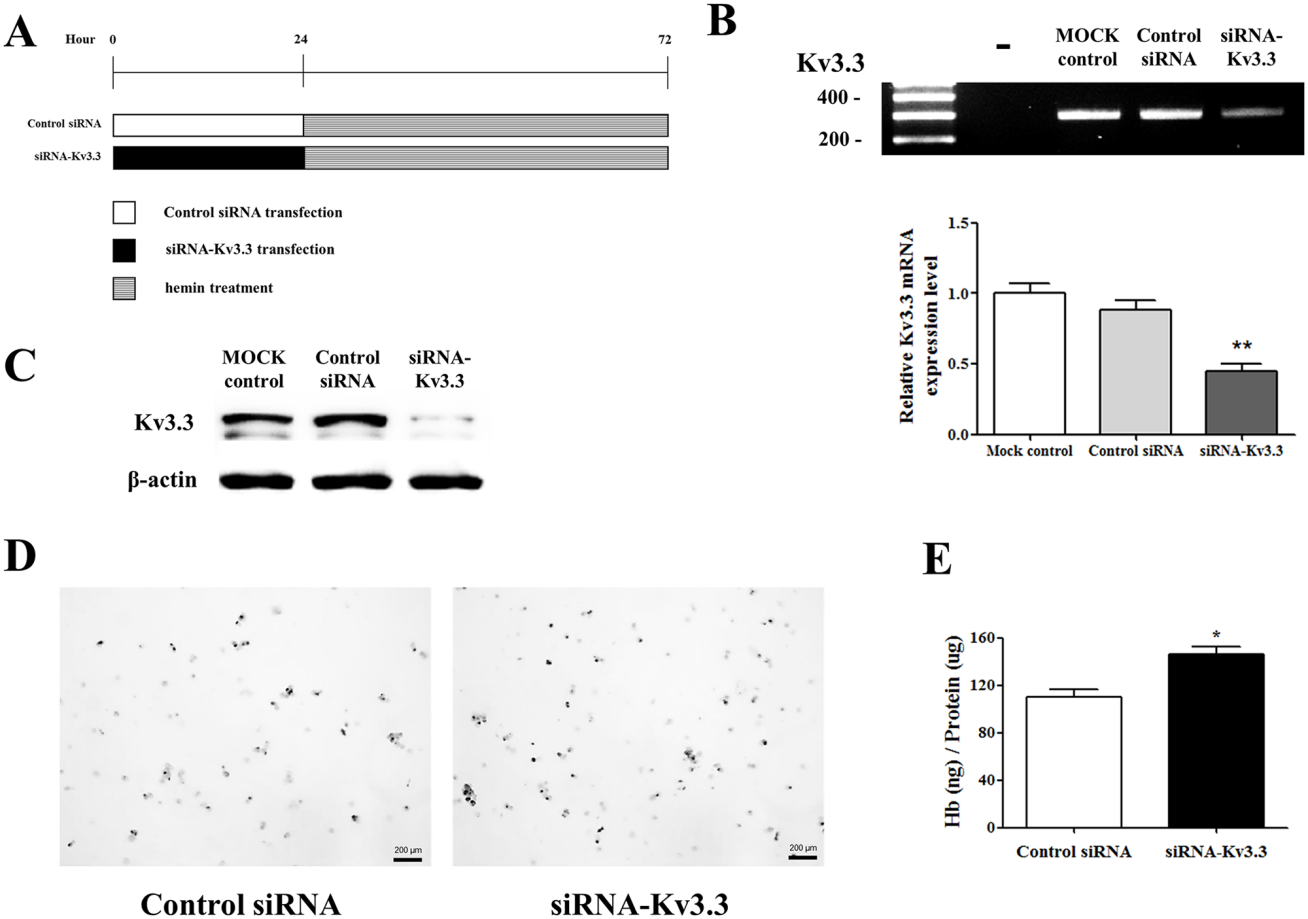
Silenced K_v_3.3 increased hemin-induced K562 cell differentiation. Hemin-induced K562 cell differentiation was significantly increased by decreasing the K_v_3.3 expression using siRNA-K_v_3.3. (A) Protocol for control siRNA and siRNA-K_v_3.3 transfection and hemin treatment. After 24 h of transfection, the K562 cells were cultured with hemin for 48 h. mRNA (B) and protein (C) expressions were suppressed when the cells were transfected with siRNA-K_v_3.3. RT-PCR was performed after 48 h of transfection. The relative mRNA expression of the K_v_3.3 was normalized to the GAPDH gene and expressed as a fold change relative to the Mock control group. (D) Benzidine staining demonstrated greater hemoglobin formation in siRNA-K_v_3.3-transfected cells (right) compared to control siRNA-transfected cells (left) during hemin-induced K562 cell erythroid differentiation (magnification ×40). Staining was performed after 24 h of transfection and 24 h of differentiation. (E) The amounts of hemoglobin content were increased by siRNA-K_v_3.3 transfection compared to control. The hemoglobin content was increased by about 50% by siRNA-K_v_3.3 transfection. Experiments were performed in triplicate, and data are expressed as mean ± standard error. *p<0.05 compared with control value.

To verify whether K_v_3.3 directly affects K562 cell differentiation, siRNA-K_v_3.3 was transfected into the K562 cells. After 24 h of transfection, the K562 cells were cultured with hemin for 48 h (Fig 3A). The degree of siRNA transfection was confirmed by RT-PCR and Western blot analysis (Fig 3B and 3C). The benzidine staining and hemoglobin quantification data demonstrated that decreased K_v_3.3 expression increased hemin-induced K562 cell differentiation (Fig 3D and 3E). The numbers of benzidine-positive cells, stained black, were increased in siRNA-K_v_3.3-transfected cells compared to the control cells. Hemoglobin quantification confirmed an approximately 50% increase in hemoglobin content following transfection with siRNA-K_v_3.3.

**Fig 4:**
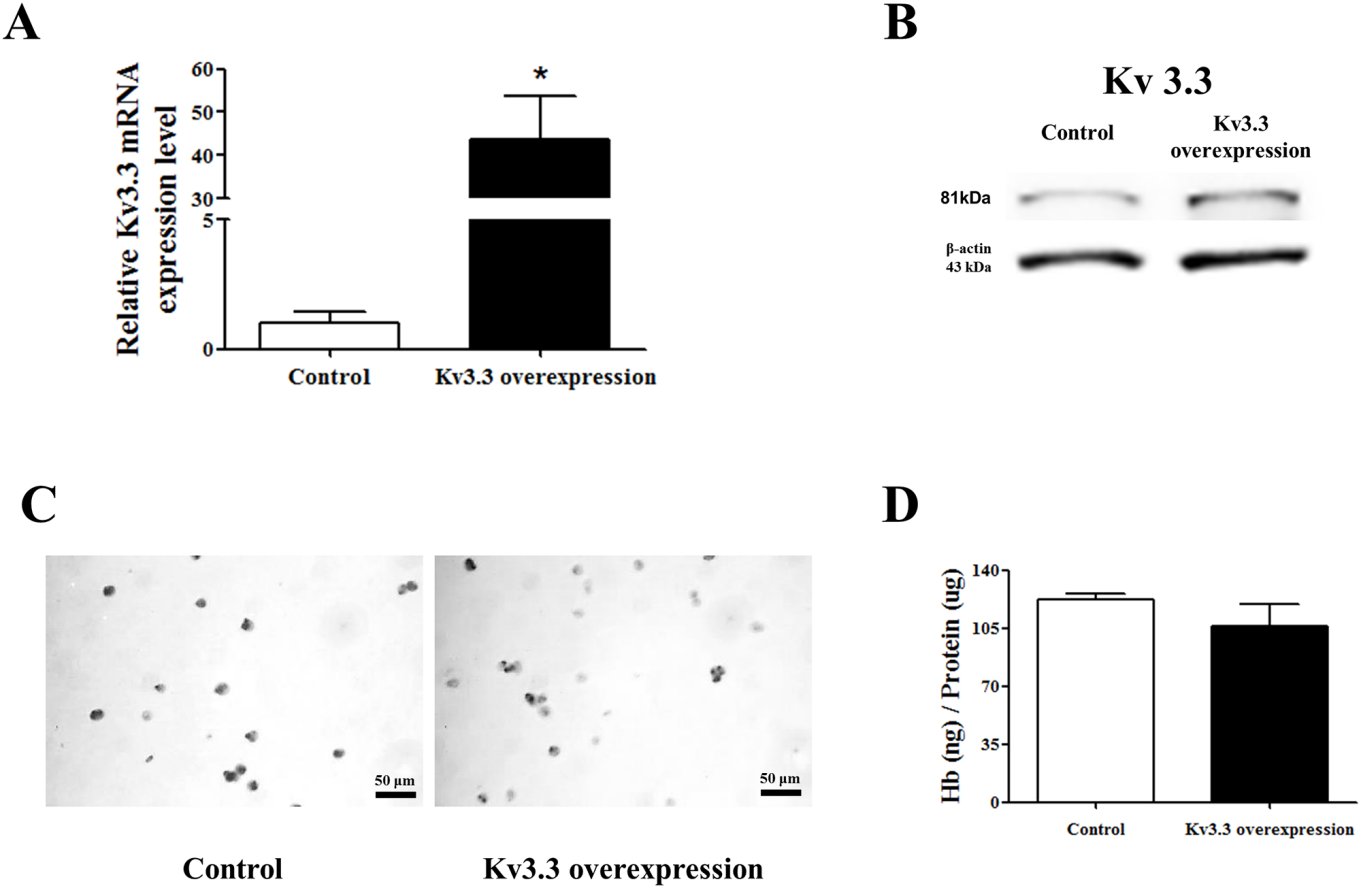
Overexpression of K_v_3.3 did not have a clear effect on hemin-induced erythroid differentiation. mRNA (A) and protein (B) expression levels of K_v_3.3 were increased in the K562 overexpressed cell line compared to the normal K562 cell line. The relative mRNA expression of the K_v_3.3 was normalized to the GAPDH gene and expressed as a fold change relative to the control group. The relative protein expressions of the Kv3.3 were expressed as a fold change relative to the control. (C) The degree of hemin-induced erythroid differentiation was measured using benzidine staining in K_v_3.3-overexpressed K562 cells or normal K562 cells. There was no significant difference between the two, however. (D) Hemoglobin quantification demonstrated that overexpressed K_v_3.3 did not have any effect on hemin-induced K562 erythroid differentiation.

Next, we established K_v_3.3-overexpressed K562 cells (Fig 4A and 4B) to verify whether K_v_3.3 overexpression inhibits hemin-induced K562 erythroid differentiation. Benzidine staining data demonstrated that the overexpression of K_v_3.3 slightly inhibited the hemin-induced K562 erythroid differentiation (Fig 4C). However, when we checked it again using hemoglobin kit assay, the data demonstrated that K_v_3.3 overexpression did not decrease the hemin-induced K562 erythroid differentiation effectively (Fig 4D), whereas the down-regulation of K_v_3.3 using siRNA- K_v_3.3 enhanced the differentiation more clearly (Fig 3E).

### 3.4. Signaling mechanisms involved in the regulation of K562 erythroid differentiation by siRNA-K_v_3.3 transfection

**Fig 5:**
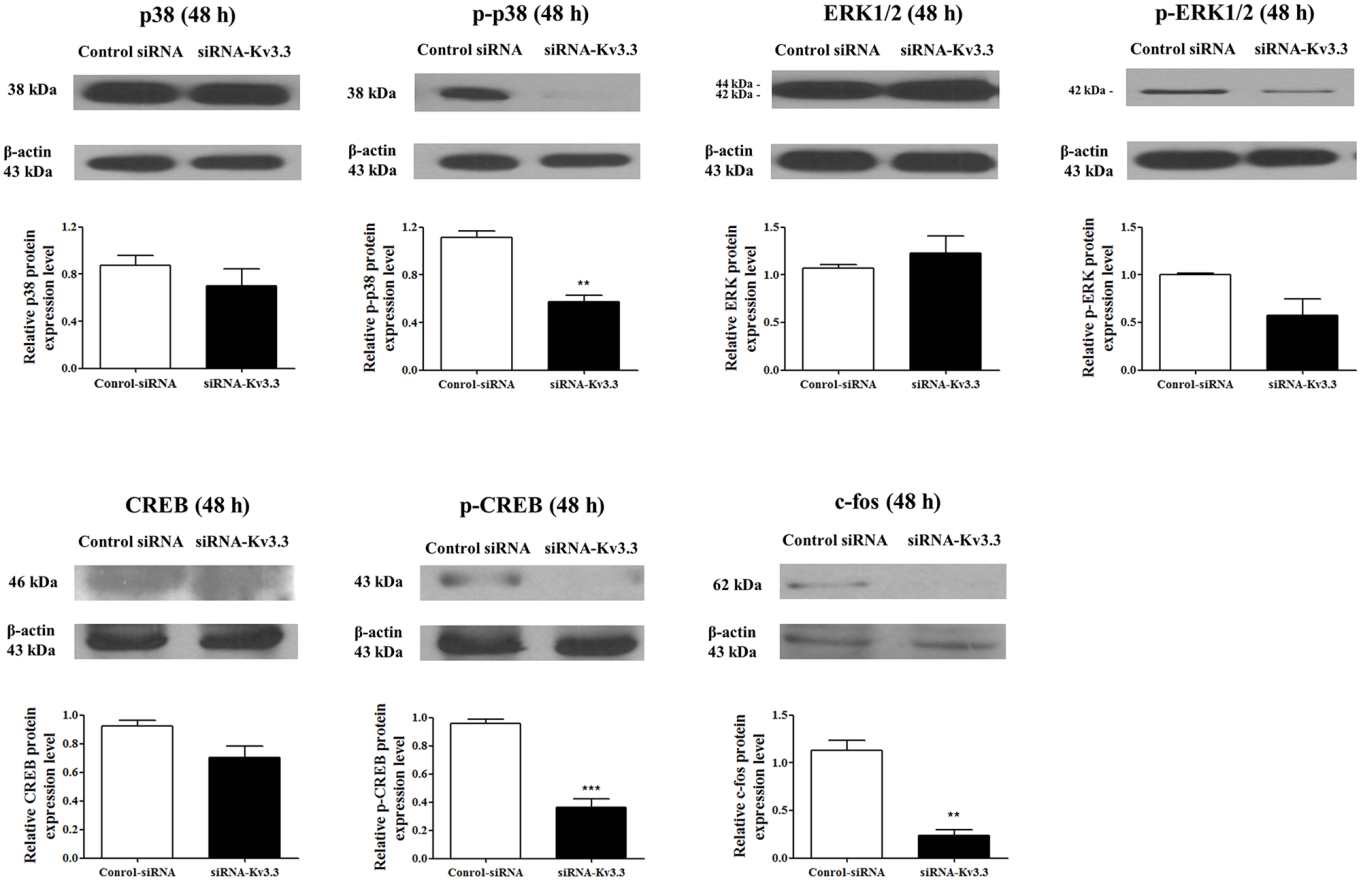
Signaling mechanisms of K562 erythroid differentiation regulation by siRNA- K_v_3.3 transfection. The expressions of p38, ERK1/2, CREB, and c-fos during cell differentiation changed with siRNA-K_v_3.3 transfection. The levels of the phosphorylated, activated forms of p38 and CREB were lower in siRNA-K_v_3.3 transfected cells than in control cells. The levels of c-fos during cell differentiation were also reduced after siRNA- K_v_3.3 transfection. The levels of phosphorylated ERK2 (p-42 MAPK) also seemed lower; however, the differences were not statistically significant. Phosphorylated ERK1 (p-44 MAPK) was not detected. No changes were noted for the total p38, total ERK1/2, and total CREB. The graphs show the quantitative analysis of each protein. Western blot assay was performed when transfected cells were differentiated for 48 h with hemin; each assay was performed in triplicate, and data are expressed as mean ± standard error. **p<0.01 compared with control value. The relative protein expressions of the signal molecules were expressed as a fold change relative to the control group.

Signal cascades involving in K562 erythroid differentiation have been well established [26–29]. We examined p38, ERK1/2, CREB, and c-fos during hemin-induced K562 erythroid differentiation in siRNA-K_v_3.3-transfected K562 cells. As shown in Fig 5, the levels of phosphorylated forms of p38 and CREB and the levels of c-fos were reduced significantly by transfection with siRNA-K_v_3.3 compared to the controls. Although the protein expression levels of the activated (phosphorylated) forms of p38 and CREB were reduced, the total amounts of p38 and total CREB were not significantly reduced. The protein expression levels of total and phosphorylated ERK1/2 (Fig 5) and the phosphorylated form of ERK2 (p-42 MAPK) also decreased; however, the difference was not statistically significant. Phosphorylated ERK1 (p-44 MAPK) was not detected in K562 cells. These changes indicated that decreased K_v_3.3 expression in K562 cells increased hemin-induced K562 cell differentiation through the reduced activation of p38, p-CREB, and c-fos.

### 3.5. K_v_ channels are not involved in the early stage of hemin-induced K562 erythroid differentiation

**Fig 6:**
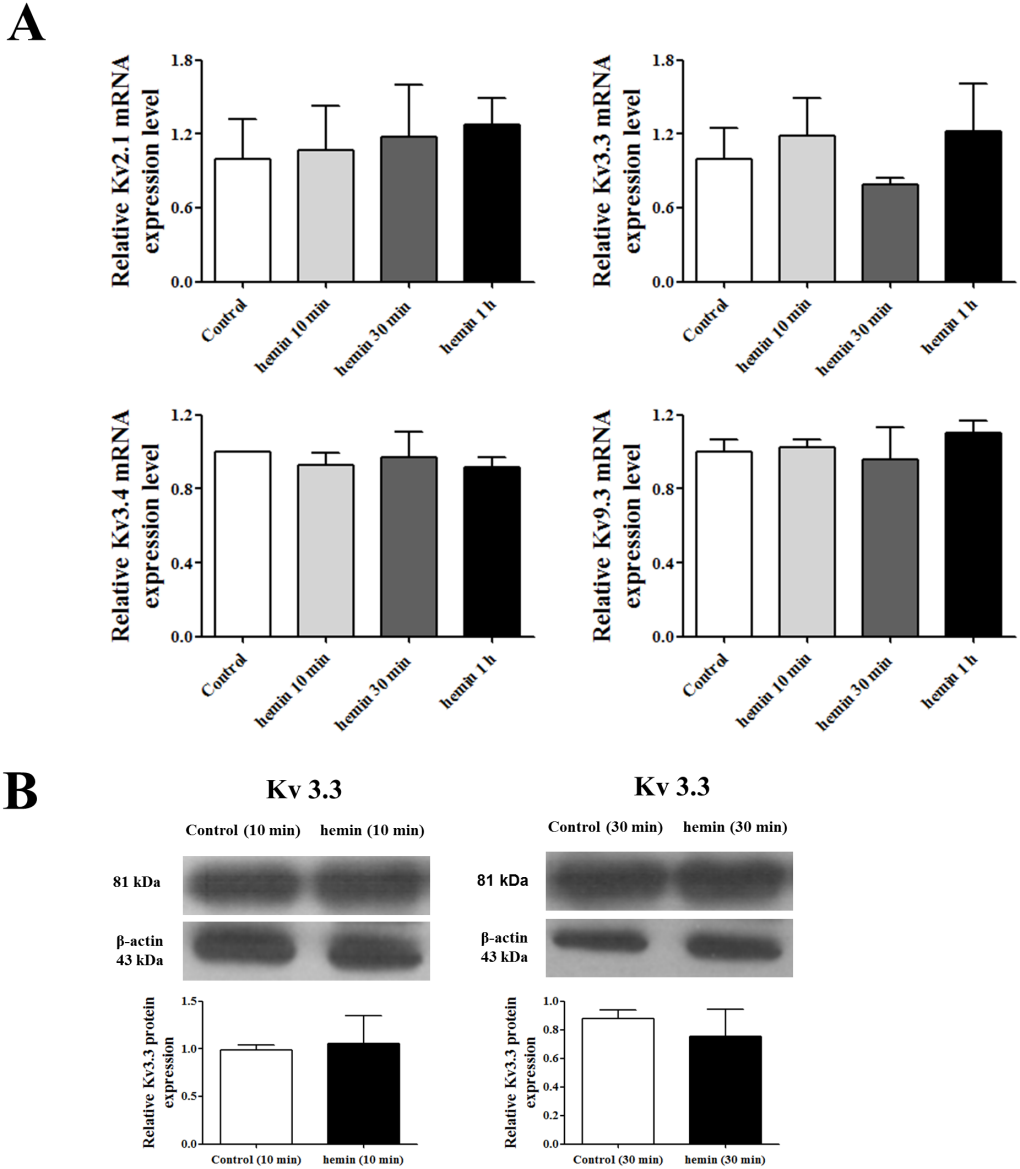
The relationship between K_v_3.3 and the early stage of K562 erythroid differentiation. (A) The mRNA expression levels of the K_v_ channels, including K_v_2.1, K_v_3.3, K_v_3.4, and K_v_9.3, did not correlate with the hemin-induced K562 erythroid differentiation at the indicated time points (10 min, 30 min, and 1 h). The relative mRNA expressions of the Kv channels were normalized to the GAPDH gene and expressed as a fold change relative to the control group. (B) The protein expression level of K_v_3.3 was estimated after inducing 10 min and 30 min of erythroid differentiation, and there was no change compared to the control cells. The relative protein expressions of the Kv3.3 were expressed as a fold change relative to the control.

**Fig 7:**
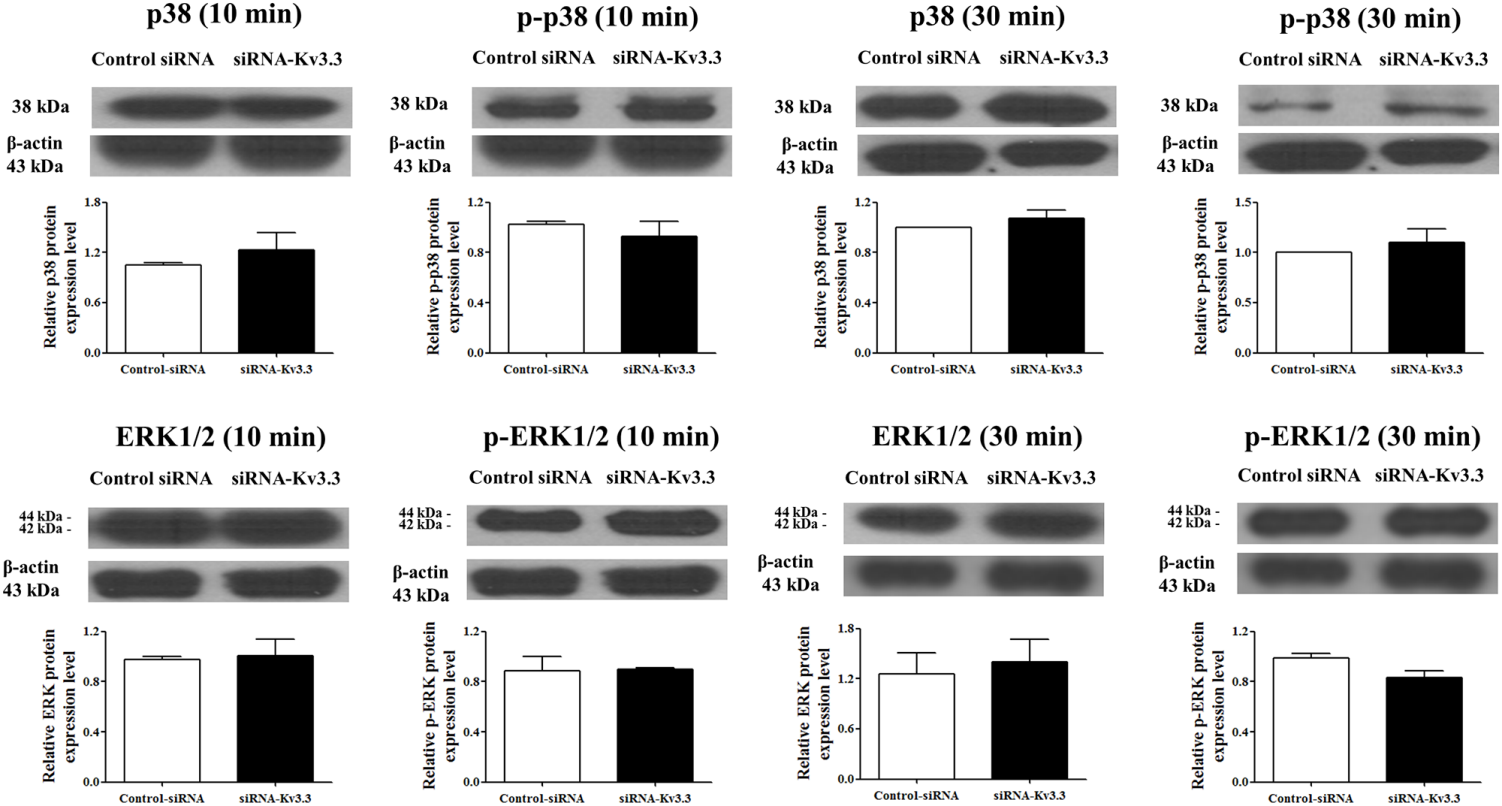
In the early stage of K562 erythroid differentiation, K_v_3.3 knockdown using siRNA-K_v_3.3 did not have any effect on the expression levels of signaling molecules involved in K562 erythroid differentiation. The protein expression levels of p38 and ERK were measured after 24 h of siRNA-K_v_3.3 transfection and 10 min or 30 min of erythroid differentiation. During the early stage of erythroid differentiation, down-regulated K_v_3.3 had no effect on the protein levels of p38 and ERK. The relative protein expressions of the signal molecules were expressed as a fold change relative to the control siRNA group.

The mRNA expression levels of K_v_2.1, K_v_3.3, K_v_3.4, and K_v_9.3 were measured at the presented time points (10 min, 30 min, and 1 h) after inducing differentiation, and we found that the expression levels of K_v_ channels including K_v_3.3 were not changed in the early stage of hemin-induced K562 erythroid differentiation (Fig 6A). The protein expression level of K_v_3.3 was also not changed after 10 min and 30 min of the erythroid differentiation (Fig 6B). K_v_3.3 knockdown using siRNA-K_v_3.3 was also performed during the early stage of hemin-induced K562 erythroid differentiation, and the transfection had no effect on the signaling mechanisms, such as p38 and ERK, which are known to be involved in the early stage of erythroid differentiation (Fig 7).

### 3.6. K_v_3.3 knockdown using siRNA-K_v_3.3 increased cell adhesion in K562 cells

**Fig 8:**
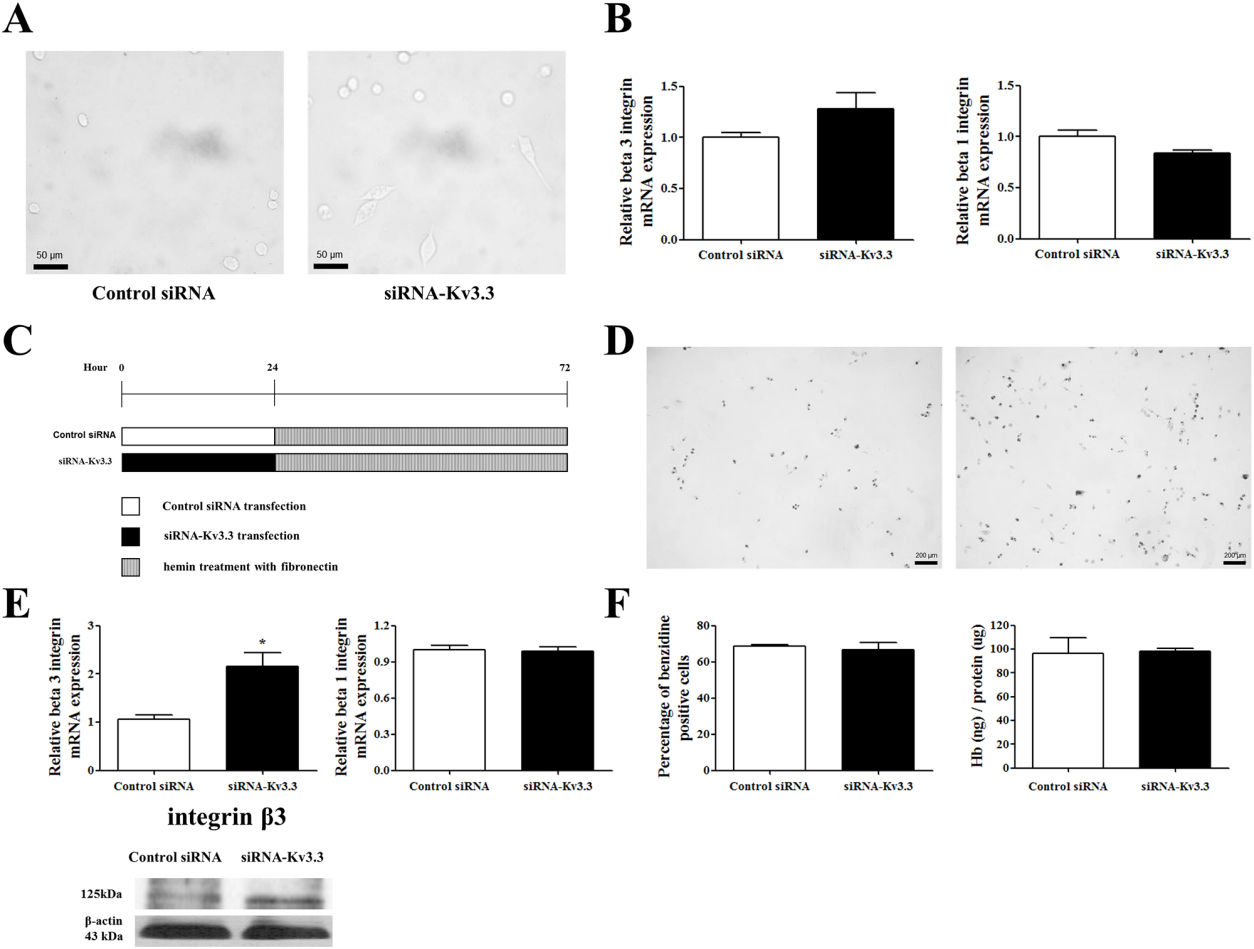
Effects of siRNA-K_v_3.3 transfection on cell adhesion. (A) Attached K562 cells were detected in siRNA-K_v_3.3-transfected cell cultures (right), whereas fewer attached cells were found in control cultures (left) (magnification ×200). (B) The mRNA expression levels of integrin β3 (left) and integrin β1 (right) during hemin-induced K562 cell erythroid differentiation by transfection of siRNA-K_v_3.3. (C) The protocol for control siRNA and siRNA-K_v_3.3 transfection and hemin treatment with fibronectin. After 24 h of transfection, the K562 cells were cultured with hemin in fibronectin-coated wells for 48 h. (D) Culturing the cells in fibronectin-coated wells (10 μg/ml) significantly improved cell adhesion during the hemin-induced erythroid differentiation of siRNA-K_v_3.3-transfected cells (magnification ×40). (E) Cells cultured in fibronectin-coated wells showed amplified effects of decreased K_v_3.3 on integrin β3 levels. The mRNA and protein expression levels of integrin β3 in siRNA-K_v_3.3-transfected cells increased much more during hemin-induced K562 cell erythroid differentiation than in the control group (*p<0.05). On the other hand, no differences were noted in integrin β1 expression between the control and siRNA-K_v_3.3 transfected-cells when the cells were cultured in fibronectin-coated wells. (F) Benzidine staining (left) and hemoglobin quantification (right) indicated that increased erythroid differentiation by siRNA-K_v_3.3 was not detected when the cells were cultured in fibronectin-coated wells. The relative mRNA expressions of the integrins were normalized to the GAPDH gene and expressed as a fold change relative to the control siRNA group.

The K562 cells transfected with siRNA-K_v_3.3 showed interesting morphological changes. Even though K562 cells are suspension and sphenoid cells, we observed the adhesion of a few K562 cells to the bottom of the 6 well plates when we incubated the cells with hemin for one or two days. The adherent cells had spindle-like shapes and more adherent cells were detected when the cells were transfected by siRNA-K_v_3.3 compared to the control cells (Fig 8A). Real time RT-PCR data demonstrated that the mRNA expression levels for integrins β3 (Fig 8B left) and β1 (Fig 8B right), which are well-known adhesion molecules, were not statistically changed by siRNA-K_v_3.3 transfection.

Next, we incubated the K562 cells in fibronectin-coated plates to enhance cell adhesion because fibronectin binds to integrins [32]. After 24 h of transfection, the K562 cells were cultured with hemin in fibronectin-coated well for 48 h (Fig 8C). As shown in Fig 8D, when siRNA-K_v_3.3 was transfected, many more cells adhered to the bottom of the plates compared to the controls (Fig 8D). In addition, the mRNA expression level of integrin β3 significantly increased in siRNA-K_v_3.3-transfected cells (Fig 8E left), whereas integrin β1 showed no difference (Fig 8E right). On the other hand, when the cells were cultured in fibronectin-coated plates with hemin, erythroid differentiation was not enhanced by siRNA–K_v_3.3 transfection. Benzidine staining and hemoglobin quantification indicated no significant differences between control and siRNA-transfected cells (Fig 8F). These results suggest that the erythroid differentiation effect induced by reduced K_v_3.3 expression in K562 cells was transformed into a cell adhesion-enhancing effect when K562 cells were provided with fibronectin.

### 3.7. Signaling mechanisms involved in the regulation of K562 differentiation by the siRNA-K_v_3.3 transfection of cells cultured in fibronectin plates

**Fig 9:**
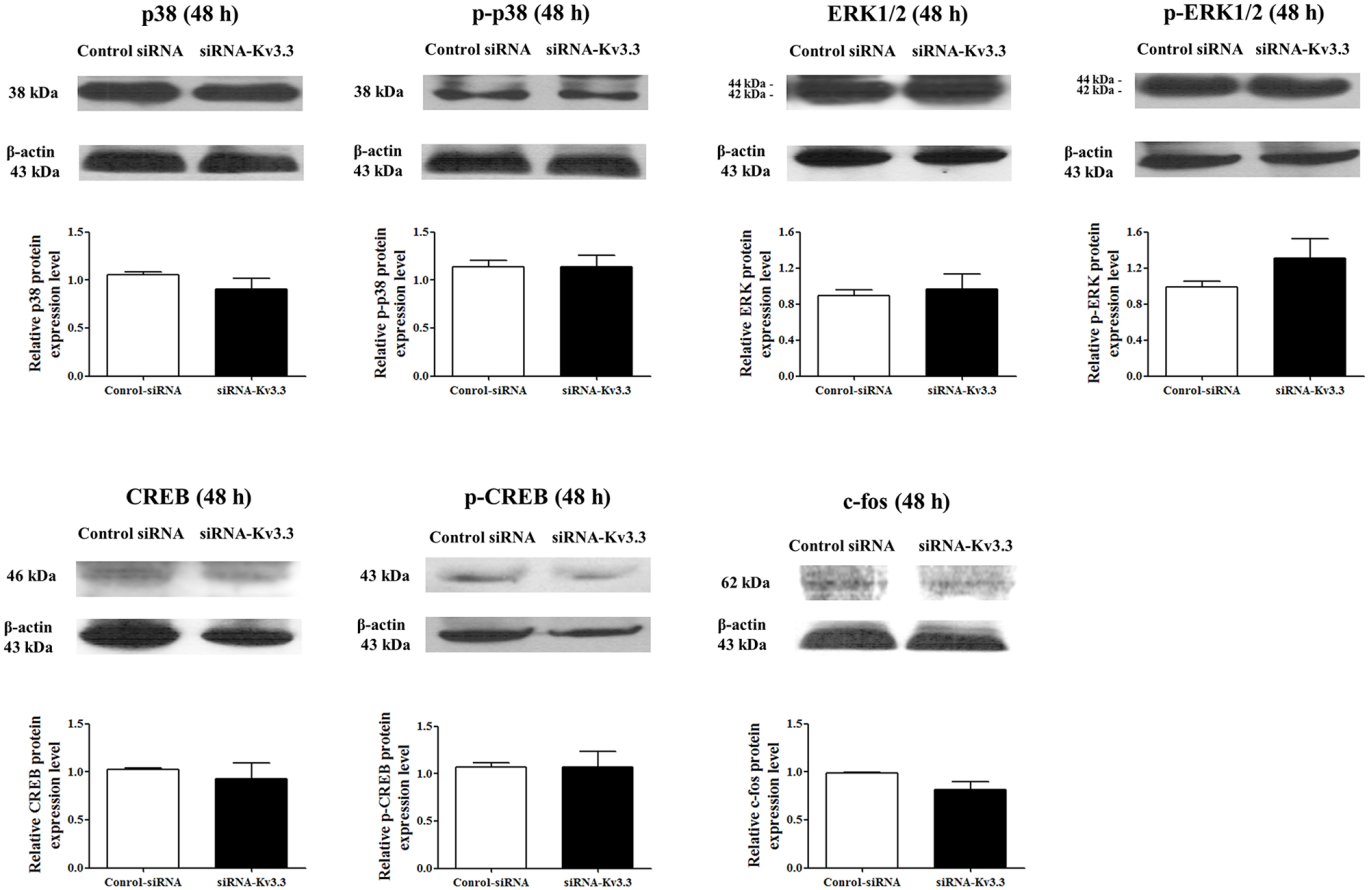
Signaling mechanisms of K562 differentiation regulation by siRNA-K_v_3.3 transfection and cultures in fibronectin plates. No changes in the expression levels of p38, ERK1/2, CREB, and c-fos were noted in response to decreased K_v_3.3 levels compared to control cells during cell differentiation in fibronectin-coated wells. No differences were noted in the total amounts or amounts of phosphorylated forms of ERK1/2, p38, and CREB expression in the control and siRNA-transfected cells. No differences were observed in the c-fos levels following siRNA-K_v_3.3 transfection when the cells were cultured with fibronectin. The graphs indicate the quantitative analysis of each protein. Western blot assays were performed when the transfected cells were differentiated for 48 h with hemin and fibronectin. Each assay was performed in triplicate, and data are expressed as mean ± standard error. The relative protein expressions of the signal molecules were expressed as a fold change relative to the control siRNA group.

Cultures in fibronectin-coated wells eliminated the K_v_3.3-mediated erythroid differentiation-inducing effect. Therefore, we compared the signaling mechanisms in the presence and absence of fibronectin. As demonstrated in Fig 9, none of the total and activated forms of ERK1/2, p38, and CREB showed any differences in the control and siRNA-transfected cells. These results are consistent with the lack of erythroid differentiation in siRNA-K_v_3.3 cells cultured in fibronectin-coated wells.

## Discussion

In the present study, we first identified which K_v_ channel subunits exist in K562 cells and the roles of K_v_ channels in K562 cell differentiation. RT-PCR and Western blot analyses demonstrated that K_v_3.3 was highly expressed in K562 cells, and that its expression level was down-regulated during the late stage of erythroid differentiation, whereas K_v_2.1 was increased and other K_v_ channels did not show any change. In K_v_3.3-silenced cells, erythroid differentiation was significantly increased during the late stage (48 h) via p-p38, p-CREB, and c-fos, whereas in the early stage of differentiation, there was no change compared to the control cells (10 min and 30 min). K_v_3.3 overexpression did not decrease the hemin-induced K562 erythroid differentiation effectively, whereas the down-regulation of K_v_3.3 using siRNA-K_v_3.3 enhanced the differentiation more clearly. According to our results, we assume that the reason K_v_3.3 overexpression did not reverse the effects of siRNA-K_v_3.3 is that K_v_3.3 is already expressed enough to regulate differentiation in native K562 cells. Interestingly, we discovered that the adhesion of K562 cells was increased in K_v_3.3-silenced cells and that adhesion was enhanced in the presence of fibronectin.

K_v_ channels have a known involvement in a range of essential cellular functions, including cell proliferation, wound healing, apoptosis, and oxygen sensing [4–9]. Cell differentiation is another important fundamental event that is regarded as having a close relationship with cell proliferation [14–16]. Several reports have demonstrated the relevance of qualitative and quantitative changes in K_v_ currents to the differentiation state of peripheral murine CD4^+^ lymphocytes [33]. More recently, K_v_ channels have been suggested to play roles in cell differentiation [34]. Wild-type *Xenopus* K_v_1.1 overexpression in *Xenopus* retinal ganglion cells results in morphological differentiation in the form of increased dendritic branching [35]. In particular, You et al. (2013) demonstrated that K_v_2.1 and K_v_3.3 may play important roles in the differentiation of human mesenchymal stem cells into adipocytes [36]. According to the results, we also could assume that K_v_2.1 and K_v_3.3 are the specific channels involved in cell differentiation in K562 cells. The function and/or expression of K_v_3.3 are closely related to K_v_2.1, and further studies need to be performed to answer the question.

To date, many voltage-dependent channels have been identified in hematopoietic stem cells, and these channels function distinctly in proliferation and differentiation [37]. The expressions of K_v_1.3 and K_v_7.1 have been identified in CD34^+^/CD45^+^/CD133^high^ cells from peripheral blood by RT-PCR. In particular, K_v_11.1, which is upregulated in leukemic hematopoietic cells, appears to be involved in the physiology of leukemic and stem cells in processes such as cell adhesion and proliferation [37].

The importance of p38, ERK1/2, CREB, and c-fos in regulating erythroid differentiation has been demonstrated. K562 erythroid differentiation is well known to involve p38, while ERK1/2 shows an opposite or no effect [26, 29, 38, 39], and CREB protein activation is involved in K562 erythroleukemia cell differentiation [29]. In the present study, the signal molecules p38, CREB, and c-fos showed a tendency to decrease during the late stage of hemin-induced erythroid differentiation due to reduced K_v_3.3. The regulation of CREB by K_v_3.3 was previously demonstrated. Tong et al. (2010) showed that the blocking of CREB reduced the expression of K_v_3.3 and c-fos in medial nucleus of the trapezoid body (MNTB) neurons [31].

Compared to what is known for other K_v_ channels, the roles of K_v_3.3 are poorly understood. Although K_v_3.3 has its own fast inactivating potassium currents when it is transfected into the HEK cell or CHO cell system [40, 41], there are few reports dealing with the electrophysiological recordings on K_v_3.3. Similar to the previous results, there is no TEA-sensitive current in K562 cells We assume that hemin-induced K562 erythroid differentiation-related K_v_ channels may be not located on cell membranes, or there would be some functions of the K_v_ channels independent from their potassium currents.

K_v_3.3 is a known oxygen-sensitive channel; it opens in the presence of oxygen and reversely closes in response to hypoxia [42]. Hypoxia reduces the production of oxygen-reactive intermediates, including H_2_O_2_, and K_v_3.3 is one of the channels that lose its fast inactivation upon external application of H_2_O_2_ [42–44]. The relationship between the function of potassium channels and oxidative stress has been well established [45], and K_v_3.3 function can be inferred to have relevance to oxidative stress. On the other hand, differentiated K562 cells produce heme contents, resulting in the increased production of reactive oxygen species [46] and naturally this causes oxidative stress. K_v_3.3 may be involved in protection against oxidative stress during erythroid differentiation, which may increase oxidative stress as a side effect. Therefore, decreases in K_v_3.3 expression in K562 cells would induce erythroid differentiation. Furthermore, expressions of signal molecules, such as MAPK and CREB, which are involve in oxidation-sensitive mechanisms [47], were altered during the hemin-induced erythroid differentiation of K562 cells transfected by siRNA-K_v_3.3.

In the present study, we detected increased adhesion properties in K_v_3.3-silenced K562 cells and found that integrins were important in the observed changes. When we used fibronectin, which interacts well with integrins, down-regulated K_v_3.3 expression during hemin-induced erythroid differentiation resulted in enhanced cell adhesion. At the same time, the hemin-induced erythroid differentiation enhancing effect of siRNA-K_v_3.3 disappeared, and no differences were seen between control and siRNA-K_v_3.3-transfected cells for expressions of the signal molecules. Järvinen et al (1993) demonstrated that differentiation inducers alter the integrin expression of K562 cells [48]. It has been demonstrated that α5β1 is the only fibronectin receptor integrin expressed in suspension-cultured K562 cells, and differentiation inducers such as TPA (12-tetradecanoyl-13-acetyl-beta-phorbol) or hemin chloride alter the expression levels of integrin; TPA increased the β3 integrin, whereas hemin chloride did not have any effect on the β3 integrin and it only decreased the β1 integrin [48]. K562 cells bind to fibronectin through the α5β1 integrin receptor when added to wells coated with fibronectin [49]. In Fig 8E, we showed that siRNA-K_v_3.3 increased β3 integrin expression when the cells were incubated in the fibronectin-coated well. From the data, we could assume that siRNA-K_v_3.3 transfection changed the original cell property of K562. In addition, from the Figs 5 and 9 we found that siRNA-K_v_3.3 transfection affected signaling pathways were changed due to the cell culture in fibronectin coated wells. It has been suggested that the Src family of tyrosine kinases (SFKs)-receptor tyrosine kinases (RTKs)-MAPK signaling is involved in integrin signaling. Fig 9 demonstrates that the transfection of siRNA-K_v_3.3 to K562 cells cultured in fibronectin-coated wells does not have any effect on the expression levels of p38 and ERK, which are MAPK families. Therefore, increased β3 integrins makes transfection of siRNA-K_v_3.3 in K562 cells affect signaling pathways other than the MAPK pathway, such as Rho-GTPase, PI3K/Akt or Rac1-related pathways [50–52]. Taken together, our data suggest that in the presence of fibronectin, the erythroid differentiation-inducing effect of a decreased K_v_3.3 expression level was changed to a cell adhesion-enhancing effect; as a result, there are no increases in hemoglobin content as a potent oxidative stress inducer. K_v_3.3 appears to enhance cell differentiation, and its enhancing effect may be regulated by providing hemin or hemin with fibronectin. Moreover, K_v_3.3 may also be involved in the cell adhesion process, similar to the function of hERG potassium channels [53], even if the effect is in the opposite direction; the hERG potassium channels enhance cell adhesion, whereas K_v_3.3 inhibits cell adhesion. Further studies are warranted to identify the particular mechanisms responsible for the different regulation of these channels.

In summary, we found several K_v_ channels in K562 cells and determined that K_v_3.3 is involved in K562 cell differentiation through signal cascades such as the MAPK, CREB, and c-fos signaling pathways. We also confirmed that K_v_3.3 is also involved in cell adhesion properties through the regulation of integrin β3. These results imply that K_v_ channels, at least K_v_3.3, function in cell differentiation processes. Therefore, further knowledge of the relationship between K_v_ channels and cell differentiation mechanisms would open a new paradigm for understanding the regulation of cell differentiation processes.

## Supporting Information

S1 Fig Effects of siRNA-K_v_3.3 transfection on the mRNA expression of K_v_2.1, K_v_3.4, and K_v_9.3 in K562 cells. The knockdown of K_v_3.3 using siRNA-K_v_3.3 increased the expression level of K_v_2.1, but it did not have any effect on the expression levels of K_v_3.4 and K_v_9.3. The relative mRNA expressions of the Kv channels were normalized to the GAPDH gene and expressed as a fold change relative to the Mock control group.
(TIF)

## Abbreviations

K_v_ channels: voltage-gated potassium channels
CREB: cAMP response element-binding protein
MAPK: mitogen activated protein kinase
ERK1/2: extracellular signal-regulated kinase 1/2
