# Supplementary figures and images for: Voltage-Gated K+ Channel, Kv3.3 Is Involved in Hemin-Induced K562 Differentiation
Source: PLoS One. 2016 Feb 5;11(2):e0148633. doi: 10.1371/journal.pone.0148633 (PMC4743930; doi:10.1371/journal.pone.0148633)

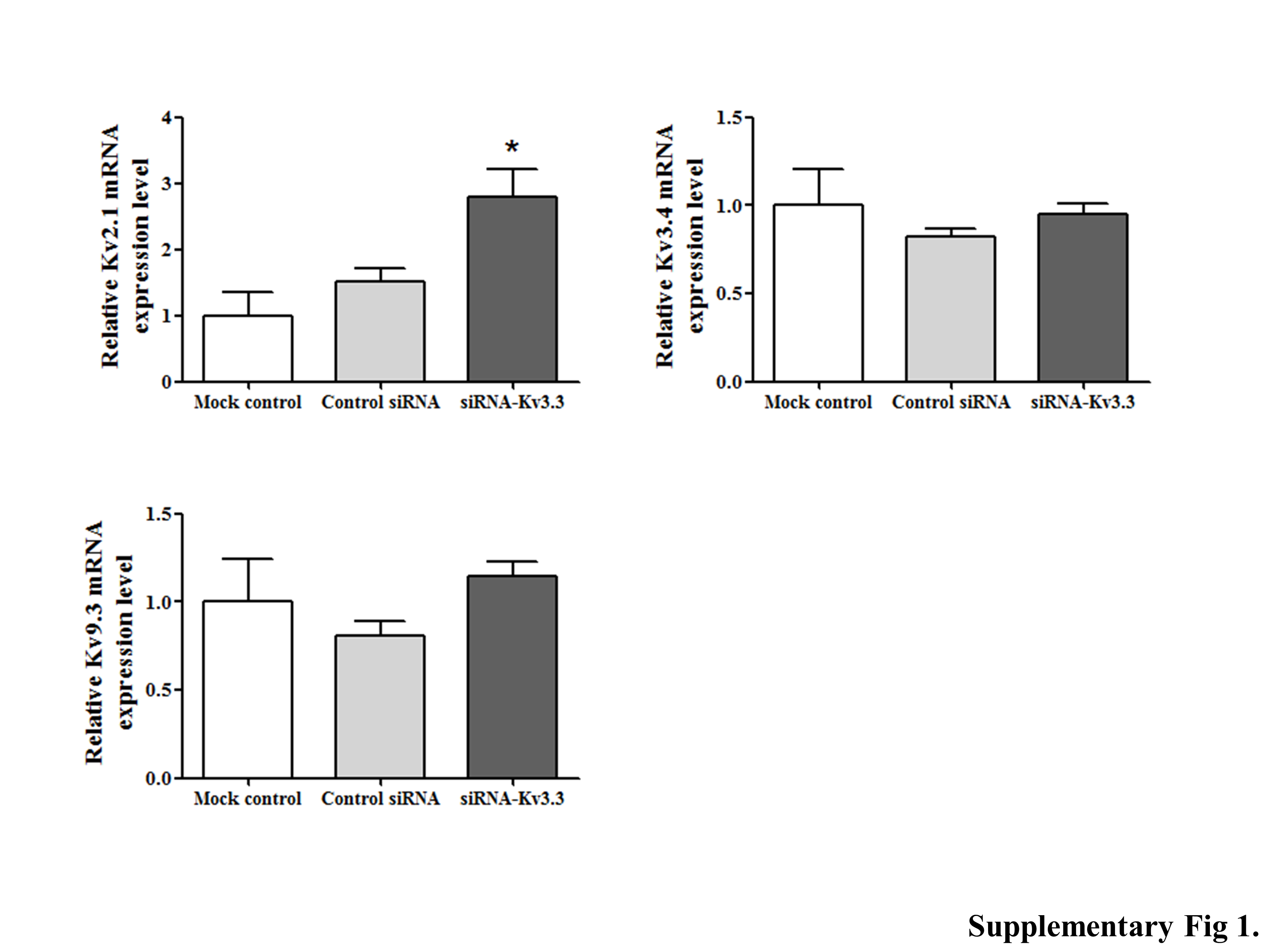

Supplement: S1 Fig — The knockdown of Kv3.3 using siRNA-Kv3.3 increased the expression level of Kv2.1, but it did not have any effect on the expression levels of Kv3.4 and Kv9.3. The relative mRNA expressions of the Kv channels were normalized to the GAPDH gene and expressed as a fold change relative to the Mock control group. (TIF) [file pone.0148633.s001.TIF]
